# Two B-C-O Compounds: Structural, Mechanical Anisotropy and Electronic Properties under Pressure

**DOI:** 10.3390/ma10121413

**Published:** 2017-12-11

**Authors:** Liping Qiao, Zhao Jin

**Affiliations:** 1 Team of Micro & Nano Sensor Technology and Application in High-altitude Regions, Xizang Engineering Laboratory for Water Pollution Control and Ecological Remediation, School of Information Engineering, Xizang Minzu University, Xianyang 712082, China; 2 School of Information Engineering, Chang’an University, Xi’an 710064, China; zhaojin@chd.edu.cn

**Keywords:** B-C-O system, stability, mechanical properties, anisotropy, electronic properties

## Abstract

The structural, stability, mechanical, elastic anisotropy and electronic properties of two ternary light element compounds, B_2_CO_2_ and B_6_C_2_O_5_, are systematically investigated. The elastic constants and phonon calculations reveal that B_2_CO_2_ and B_6_C_2_O_5_ are both mechanically and dynamically stable at ambient pressure, and they can stably exist to a pressure of 20 GPa. Additionally, it is found that B_2_CO_2_ and B_6_C_2_O_5_ are wide-gap semiconductor materials with indirect energy gaps of 5.66 and 5.24 eV, respectively. The hardness calculations using the Lyakhov-Oganov model show that B_2_CO_2_ is a potential superhard material. Furthermore, the hardness of B_6_C_2_O_5_ is 29.6 GPa, which is relatively softer and more easily machinable compared to the B_2_CO_2_ (41.7 GPa). The elastic anisotropy results show that B_6_C_2_O_5_ exhibits a greater anisotropy in the shear modulus, while B_2_CO_2_ exhibits a greater anisotropy in Young’s modulus at ambient pressure.

## 1. Introduction

It has been found that the light elements such as B, C, N, and O can form strong covalently bonded materials that show intrinsic superhard characteristics. A series of new B-C-O compounds with multifunctional properties similar to B_13_C_2_ [1,2], B_6_O [2,3], B_4_C [3], BC [4,5], B_2_C [4,6], B_3_C [4,7], BC_4_ [4], B_4_C [4], B_5_C [4], B_2_CO [8,9], and B_2_C_2_O, B_2_C_3_O, and B_2_C_5_O [10], B_4_CO_4_ [11,12], 2D B-C-O alloys [13], BC_x_N [14,15,16,17,18,19], BCN [20,21,22], BN [20,23], B_x_O [24,25,26] and C_x_N_y_ [27,28,29,30,31,32,33,34,35] has been designed by the theoretical method. These materials are almost all superhard materials. 

Using the developed particle swarm optimization (PSO) algorithm for crystal structure prediction, Wang et al. [4] explored the possible crystal structures of B-C systems that are mechanically and dynamically stable. They found that with the exception of B_4_C and BC_4_, all boron carbides have high shear moduli (more than 240 GPa), indicating their strong resistance to shape change at constant volume. Theoretical Vickers hardness calculations showed that these boron carbides are potential superhard materials because the predicted hardnesses exceed 40 GPa. Zhang et al. [20] designed different kinds of superhard materials using the Crystal structure AnaLYsis by Particle Swarm Optimization (CALYPSO) algorithm. They found superhard phases in binary B-N compounds (such as Pct-BN, *Pbca*, Z-BN, BC_8_-BN, and M-BN) and ternary B-C-N compounds (such as *I*-4*m*2 BCN, *Imm*2 BCN [21], and *P*3*m*1 BCN [22]). *Pbca*-BN with an orthorhombic structure was investigated by first-principles calculations by Fan et al. [23], who found that *Pbca*-BN has the shear modulus of 316 GPa, bulk modulus of 344 GPa, large Debye temperature of 1734 K, and hardness of 60.1 GPa. Ternary B-C-N compounds *I*-4*m*2 BCN, *Imm*2 BCN, and *P*3*m*1 BCN were investigated by first-principles calculations by Fan et al. [21,22] and were all found to be potential superhard materials. Electronic structure studies showed that BCN materials in the *P*3*m*1 and *I*-4*m*2 phases are indirect semiconductors with band gaps of 4.10 eV and 0.45 eV, respectively, while the *Imm*2 phase is a direct semiconductor with a band gap of 2.54 eV. 

Recently, Zhang et al. [10] performed extensive structure searches to explore the potential energetically stable B_2_C*x*O (*x* ≥ 2) phases at ambient pressure using the current developed CALYPSO algorithm. B_2_C_2_O, B_2_C_3_O, and B_2_C_5_O in the tetragonal *I*4_1_/*amd*, *I*-4*m*2, and *P*-4*m*2 phases, respectively, were reported in ref. [10]. The phonon dispersion and formation enthalpy calculations revealed that B_2_C_2_O, B_2_C_3_O, and B_2_C_5_O are all dynamically stable and can be synthesized at ambient conditions. The hardnesses of B_2_C_2_O, B_2_C_3_O, and B_2_C_5_O are 57, 62, and 68 GPa using Gao’s model [36], and they are all potential superhard materials. B_2_CO, a potential superhard material in the B-C-O system, can stably exist in several different phases. tP4-B_2_CO, with the *P*-4*m*2 phase and tI16-B_2_CO with the *I-*42*d* phase have been reported by Li et al. [9], while oP8-B_2_CO with the *Pmc*2_1_ phase was systematically investigated by Liu et al. [8] The elastic constants, phonon dispersion spectra and formation enthalpies confirmed the mechanical, dynamical and thermodynamic stabilities, respectively, of oP8-B_2_CO, tP4-B_2_CO, and tI16-B_2_CO. Calculation of the Vickers hardness of oP8-B_2_CO shows that oP8-B_2_CO is a superhard material, and the Vickers hardness of oP8-B_2_CO (47.70 GPa) is much closer to tP4-B_2_CO (49.24 GPa) and tI16-B_2_CO (49.56 GPa). The band structure calculations illustrate that all B_2_CO phases are semiconductor materials with indirect band gaps, and oP8-B_2_CO has the widest band gap (3.540 eV), greater than those of tP4-B_2_CO (1.658 eV) and tI16-B_2_CO (2.988 eV). 

B_4_CO_4_, found in the *I*-4 space group, is a novel tetragonal thermodynamically stable phase, and two low-enthalpy metastable compounds (B_6_C_2_O_5_ in *P*1 phase, B_2_CO_2_ in *C*2/*m* phase) have been discovered by Wang et al. [12] using the widely used and evolutionary Universal Structure Predictor: Evolutionary Xtallography (USPEX) algorithm. The structural, stability, mechanical, elastic anisotropy and electronic properties of B_4_CO_4_ have been systematically investigated as described in ref. [11,12]. However, the structural, stability, mechanical, and elastic anisotropy properties of B_2_CO_2_ and B_6_C_2_O_5_ have not been reported. Therefore, in this work, we systematically investigate the structural, stability, mechanical, elastic anisotropy and electronic properties of B_2_CO_2_ and B_6_C_2_O_5_.

## 2. Theoretical Methods

The calculations were performed using density functional theory with the exchange-correlation functional treated using the generalized gradient approximation (GGA) in the Perdew-Burke-Ernzerhof (PBE) form [37] and the Perdew-Burke-Ernzerhof for solids (PBEsol) form [38], and with local density approximation (LDA) based on the data of Ceperley and Alder as parameterized by Perdew and Zunger (CA-PZ) [39,40]. The calculations in this work were performed using the Cambridge Serial Total Energy Package (CASTEP) code [41]. Structural optimizations were conducted using the Broyden-Fletcher-Goldfarb-Shanno (BFGS) minimization algorithm [42]. A high-density *k*-point [43] sampling with a grid spacing of less than 2π × 0.025 Å^−1^ (4 × 16 × 7 for B_2_CO_2_, 9 × 9 × 9 for B_2_CO_2_) in the Brillouin zone was used. The self-consistent convergence criterion for the total energy was 5 × 10^−6^ eV/atom, the maximum force on the atom was 0.01 eV/Å, the maximum ionic displacement was within 5 × 10^−4^ Å and the maximum stress was within 0.02 GPa. The HSE06 hybrid functional [44] was used for the calculations of electronic structures of B_2_CO_2_ and B_6_C_2_O_5_.

## 3. Results and Discussion

### 3.1. Structural Properties

**Figure 1:**
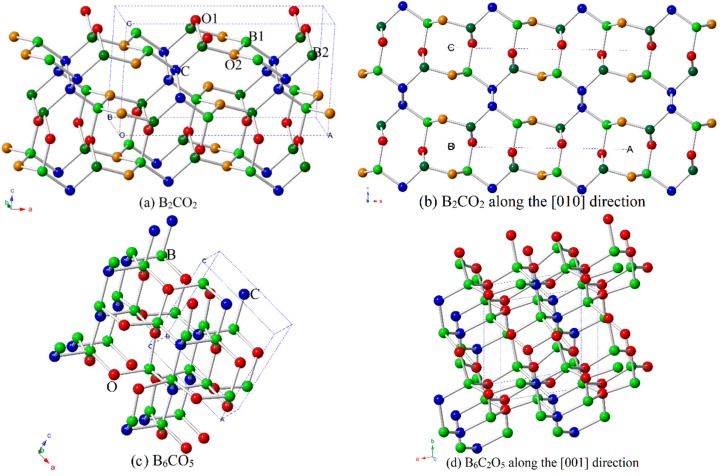
Lattice structure of B_2_CO_2_ (**a**); and along the [010] direction (**b**); and lattice structure of B_6_C_2_O_5_ (**c**); and along the [001] direction (**d**).

The crystal structures of B_2_CO_2_ and B_6_C_2_O_5_ are shown in Figure 1a–d, respectively. For the structure shown in Figure 1a, there are two inequivalent oxygen atom positions (O1 in (0.1337, 0.0, 0.0474), red; O2 in (0.9735, 0.5, 0.2763), orange), two inequivalent boron atom positions (B1 in (0.8891, 0.0000, 0.2433), light green; B2 in (0.1298, 0.5000, 0.2027), dark green), and an equivalent carbon atom (C in (0.2497, 0.0000, 0.5855), blue) in B_2_CO_2_. In the structure shown in Figure 1c, each atom occupies a different position in B_6_C_2_O_5_ (B in (0.4165, 0.5981, 0.0121), (0.6796, 0.1566, 0.1255), (0.9807, 0.7214, 0.2895), (0.5464, 0.8782, 0.5674), (0.3288, 0.3515, 0.4784) and (0.1293, 0.0127, 0.8430); C in (0.4451, 0.9082, 0.8863) and (0.3015, 0.6221, 0.3228); and O in (0.5669, 0.1822, 0.4377), (0.8809, 0.8281, 0.5836), (0.7350, 0.4833, 0.0788), (0.9854, 0.0320, 0.1394), and (0.2105, 0.3326, 0.7576)). The calculated lattice parameters of B_2_CO_2_, B_6_C_2_O_5_, and other B-C-O compounds are listed in Table 1. The calculated lattice parameters of B_2_CO_2_ and B_6_C_2_O_5_ are in excellent agreement with the previous report [12], and the calculated lattice parameters of the other B-C-O compounds are also in excellent agreement with the previous report (see Table 1). 

**Figure 2:**
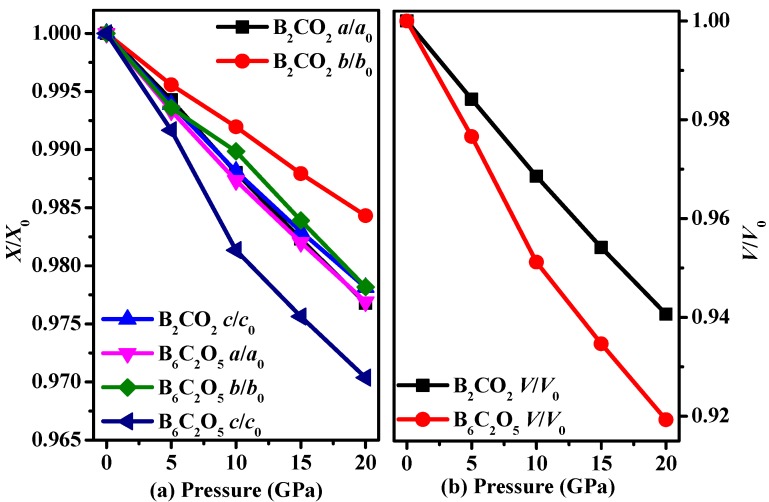
Lattice constants *a*/*a*_0_, *b*/*b*_0_, *c*/*c*_0_ compression as functions of pressure for B_2_CO_2_ and B_6_C_2_O_5_ (**a**); and primitive cell volume *V*/*V*_0_ for B_2_CO_2_ and B_6_C_2_O_5_ (**b**).

The relationships among the calculated lattice parameter ratios X/X_0_ (a/a_0_, b/b_0_, c/c_0_) and lattice volume ratios V/V_0_ of B_2_CO_2_ and B_6_C_2_O_5_ and pressure are shown in Figure 2, where a_0_, b_0_, c_0_ and V_0_ are the zero temperature and zero pressure equilibrium lattice constants and lattice volume, respectively. For B_2_CO_2_, it can be easily seen that the compression of the *b*-axis is the most difficult, whereas that of the *a*-axis is the easiest. For B_6_C_2_O_5_, similar to B_2_CO_2_, the *b*-axis is the most difficult to compress, while the *c*-axis is the easiest to compress. When the pressure increases, the compression along the *b*-axis of B_6_C_2_O_5_ is much larger than that along the *b*-axis of B_2_CO_2_. In addition, it is found that the lattice constants b/b_0_, c/c_0_ compression of B_6_C_2_O_5_ is changed at 10 GPa. This is because the lengths of some bonds decrease strongly along the lattice vector *b*-axis or the lattice vector *c*-axis. For example, there are three kinds of B-O bonds along the lattice vector *c*-axis; the bond lengths of B-O bonds are 1.571 Å (1.553 Å), 1.456 Å (1.459 Å) and 1.561 Å (1.543 Å) at 0 (5) GPa, respectively, and while the lengths of the first and the third bonds suddenly decreased to 1.516 Å and 1.508 Å under 10 GPa, the second bond length suddenly decreased to 1.489 Å under 10 GPa. Under 15 GPa, the first, second and third bond lengths dropped to 1.502 Å, 1.479 Å and 1.497 Å, respectively. From the above discussion, we can see that the abrupt change of the lattice constants c/c_0_ compression of B_6_C_2_O_5_ is due to the sudden decrease of the bond length of the B-O bonds along the lattice vector *c*-axis. The abrupt changes along the lattice vector *b*-axis is similar to that due to the abnormal change of the B-C bond length and the B-O bond. Their lattice constants changes show different trends with the pressure, which is related to the crystal structure and atomic composition. B_6_C_2_O_5_ exists in the P1 space group, whereas B_2_CO_2_ is found in the C2/m space group and the P1 space group has the lowest symmetry among the 230 space groups. In Figure 2b, for the lattice volume ratio, we predict that B_2_CO_2_ has better compressive resistance than B_6_C_2_O_5_. The results of Figure 2b confirm our prediction. Here, we can also predict that the bulk modulus of B_2_CO_2_ is greater than that of B_6_C_2_O_5_.

### 3.2. Stability

**Figure 3:**
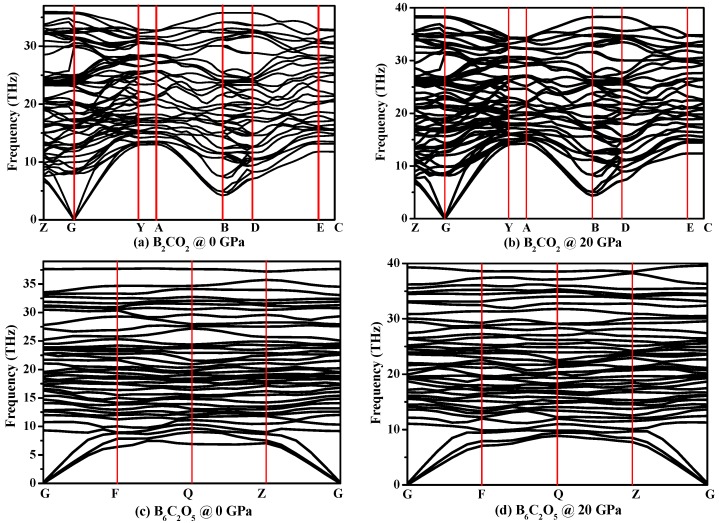
Phonon spectra of B_2_CO_2_ and B_6_C_2_O_5_ under different pressures: B_2_CO_2_ @ 0 GPa (**a**); B_2_CO_2_ @ 20 GPa (**b**); B_6_C_2_O_5_ @ 0 GPa (**c**); and B_6_C_2_O_5_ @ 20 GPa (**d**).

To demonstrate the dynamical stability of these compound, their phonon spectra are shown in Figure 3. At ambient pressure, there are no virtual frequencies in the entire Brillouin zone, proving that B_2_CO_2_ and B_6_C_2_O_5_ are both dynamically stable. When *P* = 20 GPa, there are still no virtual frequencies in the entire Brillouin zone; in other words, B_2_CO_2_ and B_6_C_2_O_5_ are still dynamically stable. The calculated elastic constants of B_2_CO_2_ and B_6_C_2_O_5_ under different pressures are listed in Table 2. The calculated elastic constants of B_2_CO_2_ and B_6_C_2_O_5_ at ambient pressure are in excellent agreement with the previously reported results [12]. There is no doubt that based on the data presented in Table 2, the elastic constants of B_2_CO_2_ and B_6_C_2_O_5_ satisfy the mechanical stability criteria [45], indicating that B_2_CO_2_ and B_6_C_2_O_5_ are mechanically stable. While dynamical and mechanical stabilities are verified by the phonon spectra and elastic constants, respectively, the formation enthalpy can be used to determine whether the new materials can be synthesized experimentally. Wang et al. have also described the possible synthetic routes of B_2_CO_2_ and B_6_C_2_O_5_ in Ref. [12]. With regard to elastic constants, compared to other B-C-O compounds, some elastic constants of B_2_CO_2_ and B_6_C_2_O_5_ are larger and some are smaller. 

### 3.3. Mechanical Properties and Elastic Anisotropy

**Figure 4:**
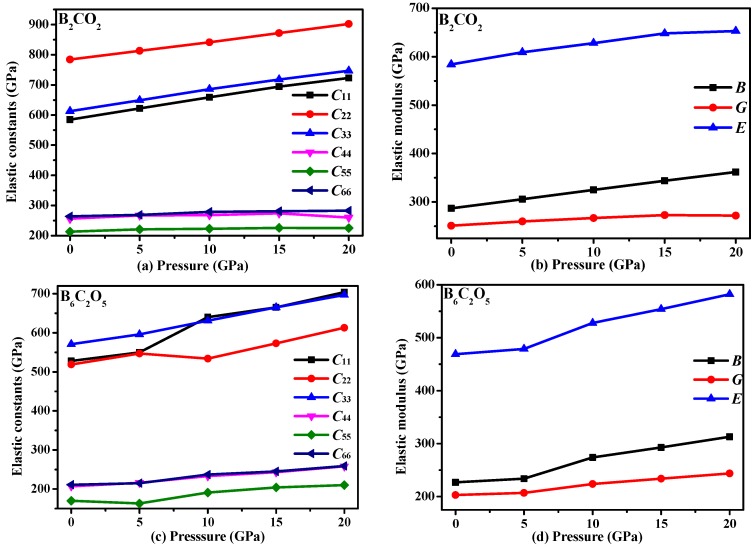
Elastic constants (**a**) and elastic modulus (**b**) as functions of pressure for B_2_CO_2_; and Elastic constants (**c**) and elastic modulus (**d**) as functions of pressure for B_6_C_2_O_5_.

The primary elastic constants and elastic modulus values of B_2_CO_2_ and B_6_C_2_O_5_ as functions of pressure are shown in Figure 4, and the elastic modulus values for B_2_CO_2_, B_6_C_2_O_5_ and other B-C-O compounds are also listed in Table 2. Inspection of Figure 4 shows that almost all primary elastic constants and elastic moduli increase with increasing pressure. For B_2_CO_2_, the rate of increase of the elastic constants and elastic moduli remains almost constant, while for B_6_C_2_O_5_, the elastic constants and elastic moduli suddenly increase from 5 GPa to 10 GPa, and the rate of increase of elastic constants and elastic moduli remains almost constant under other pressures. As is well-known, the elastic constant represents the elastic properties of a material. Elastic constants provide a description of the relationship of stress and strain of different directions in an anisotropic medium. The elastic constants also obey Hooke’s law, and strain is proportional to stress, as expressed by Hooke’s law *F* = −*kx*, where *k* is a constant, called the stiffness coefficient. The stiffness coefficient is a complex physical quantity related to the material itself and the external conditions such as the temperature, so that the changes in the elastic constants for B_6_C_2_O_5_ observed in Figure 4c are also understandable. The lattice constant changes show different trends with the pressure, which are related to the differences in the crystal structure and atomic composition. B_6_C_2_O_5_ is found in the *P*1 space group, while B_2_CO_2_ is found in the *C*2/*m* space group, and the *P*1 space group has the lowest symmetry among the 230 space groups. For the *P*1 space group, there are 21 independent elastic constants, while for the *C*2/*m* space group, there are only 13 independent elastic constants. The hardnesses of B_2_CO_2_ and B_6_C_2_O_5_ were calculated by Wang et al. [11] using the Chen-Niu model [47] and the Lyakhov-Oganov model [48]. The results show that B_2_CO_2_ is a kind of superhard material. The Young’s modulus *E* is calculated as: *E* = 9*BG*/(3*B* + *G*). The calculated results for Young’s modulus for B_2_CO_2_ and B_6_C_2_O_5_ are also listed in Table 2. The Young’s moduli of B_2_CO_2_ and B_6_C_2_O_5_ increase with pressure, and the increase for B_6_C_2_O_5_ is greater than that for B_2_CO_2_. The increase for B_6_C_2_O_5_ was 23.88%, almost two times larger than that of B_2_CO_2_ (12.01%). 

**Figure 5:**
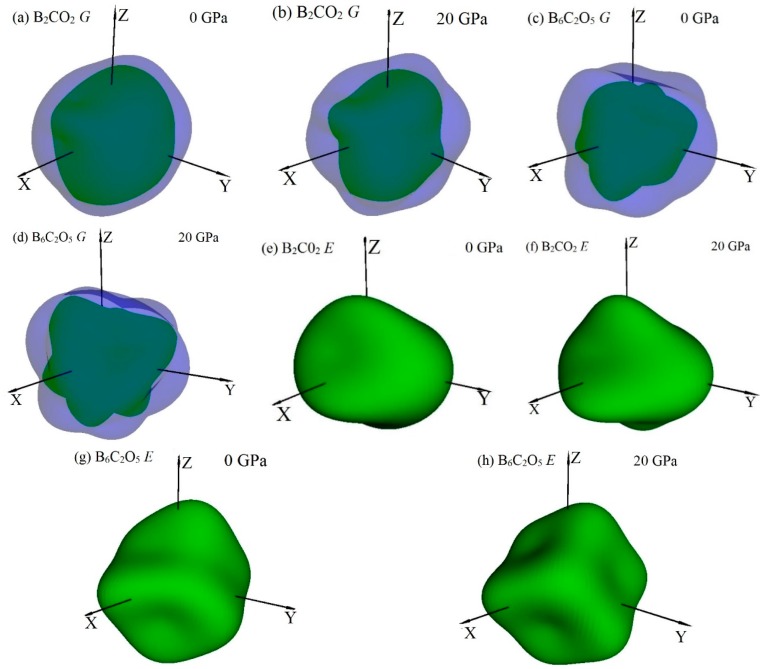
Directional dependence of the shear modulus at 0 GPa (**a**) and 20 GPa (**b**), and Young’s modulus at 0 GPa (**c**) and 20 GPa (**d**) of B_2_CO_2_; and Directional dependence of the shear modulus at 0 GPa (**e**) and 20 GPa (**f**), and Young’s modulus at 0 GPa (**g**) and 20 GPa (**h**) of B_6_C_2_O_5_.

**Figure 6:**
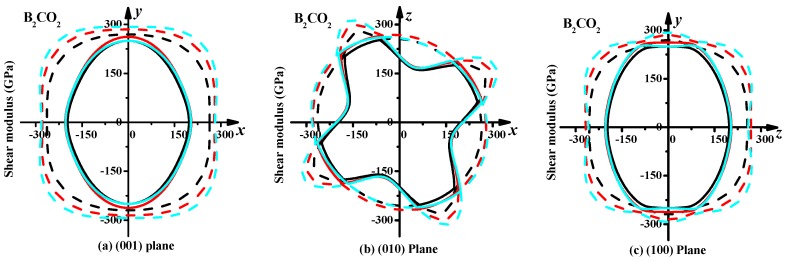

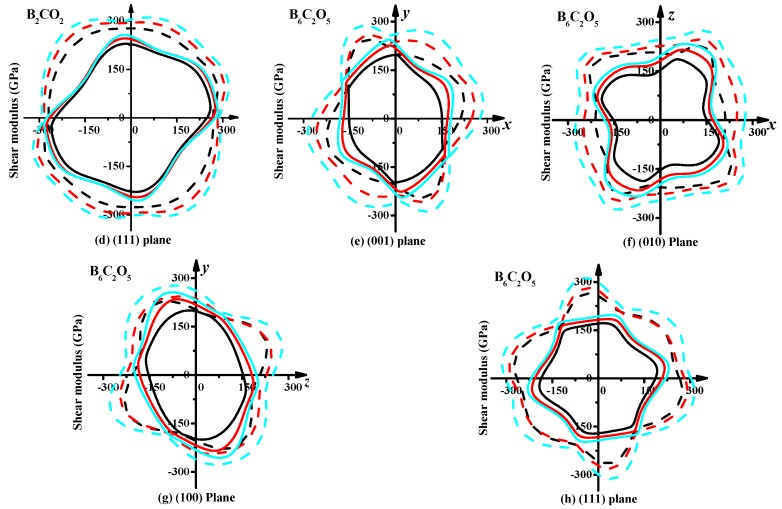
Planar projections of the directional dependence of the shear modulus in B_2_CO_2_, (**a**): (001) plane, (**b**): (010) plane, (**c**): (100) plane, and (**d**): (111) plane; and Planar projections of the directional dependence of the shear modulus in B_6_C_2_O_5_, (**e**): (001) plane, (**f**): (010) plane, (**g**): (100) plane, and (**h**): (111) plane.

**Figure 7:**
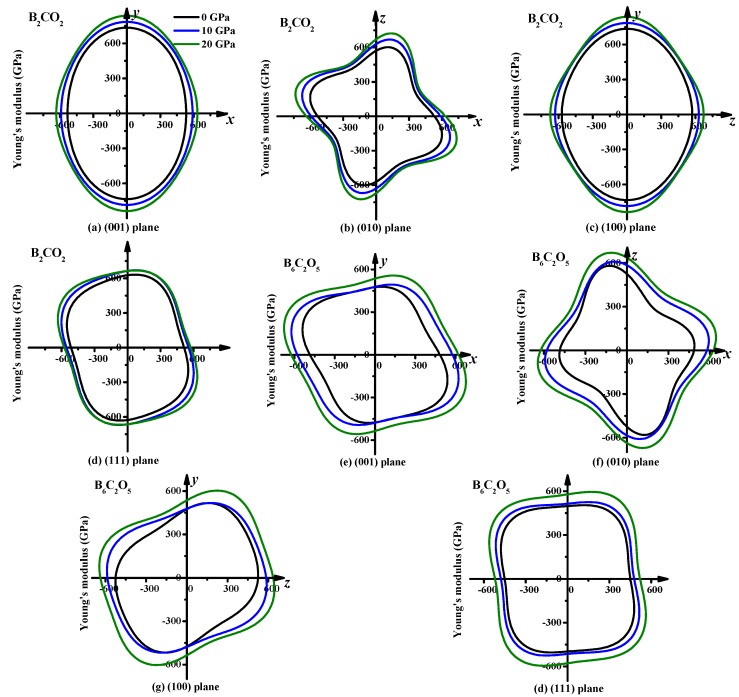
Planar projections of the directional dependence of the Young’s modulus in B_2_CO_2_, (**a**): (001) plane, (**b**): (010) plane, (**c**): (100) plane, and (**d**): (111) plane; and Planar projections of the directional dependence of the Young’s modulus in B_6_C_2_O_5_, (**e**): (001) plane, (**f**): (010) plane, (**g**): (100) plane, and (**h**): (111) plane.

To analyze the anisotropy of B_2_CO_2_ and B_6_C_2_O_5_ more systematically, the anisotropies of the shear moduli and Young’s moduli of B_2_CO_2_ and B_6_C_2_O_5_ are investigated using the ELAM codes [22,45,49]. To better understand the mechanical and anisotropic properties of B_2_CO_2_ and B_6_C_2_O_5_, 3D surface figures of the shear moduli and Young’s moduli for B_2_CO_2_ and B_6_C_2_O_5_ are shown in Figure 5. The 3D surface figures represent the geometric figure that consists of the maximum or minimum value of the shear modulus or Young’s modulus in all directions of space. The magnitude of the shear modulus or Young’s modulus in all directions can be studied by using plane cutting, and the magnitude of the shear modulus or Young’s modulus in any plane can be represented by a two-dimensional graph. The 2D representations of the shear modulus and Young’s modulus are shown in Figure 6 and Figure 7, respectively. The 3D figure appears as a spherical shape for an isotropic material, while the deviation from the spherical shape is a measure of the degree of anisotropy [50]. It is clear that the shear and Young’s moduli of B_2_CO_2_ and B_6_C_2_O_5_ exhibit different degrees of anisotropy, and the anisotropies of the shear and Young’s moduli of B_2_CO_2_ and B_6_C_2_O_5_ increase with as the pressure increases from 0 GPa to 20 GPa. For example, a depression appears on the minimum value surface (green surface) of the shear modulus of B_2_CO_2_ at 20 GPa (see Figure 5b), but there is no dent under ambient pressure. In another example, at 20 GPa, a distinct dent appears on the three-dimensional Young’s modulus surface for B_6_C_2_O_5_ (see Figure 5h). Similarly, the indentation on the three-dimensional Young’s modulus surface for B_6_C_2_O_5_ is not so obvious at ambient pressure.

The sectional profiles are constructed on the basis of analysis of the geometrical characteristics of the 3D surfaces of the shear and Young’s moduli of B_2_CO_2_ and B_6_C_2_O_5_. Sectional drawings of the shear and Young’s moduli are shown in Figure 6 and Figure 7, respectively. The black, red and cyan lines represent the shear moduli at 0, 10 and 20 GPa, while the dash-dotted line and the solid line represent the maximum and minimum values of the shear modulus along different directions in the (001) plane (*xy* plane), (010) plane (*xz* plane), (100) plane (*yz* plane) and (111) plane, respectively. As shown in Figure 6, the anisotropy of B_2_CO_2_ and B_6_C_2_O_5_ in the shear modulus increases with increasing pressure. Examination of Figure 6a shows that along the direction of the *x*-axis, the maximum values of shear modulus shrink inwards at 20 GPa, but the maximum values of the shear modulus remain constant in the direction of the *x*-axis at ambient pressure. According to the geometrical shape of the profile, the anisotropy of B_6_C_2_O_5_ in the shear modulus is larger than that of B_2_CO_2_ at ambient pressure, while under high pressure, the anisotropy of B_6_C_2_O_5_ is smaller than that of B_2_CO_2_. This can be proved by comparing the ratio of the maximum to the minimum of the shear modulus (*G*_max_/*G*_min_). The maximum and the minimum values of the shear modulus and Young’s modulus for B_2_CO_2_ and B_6_C_2_O_5_ are listed in Table 3. The maximum value of shear modulus for B_2_CO_2_ increases with pressure, while the minimum value of the shear modulus for B_2_CO_2_ increases first and then decreases with pressure, so that the anisotropy of B_2_CO_2_ in the shear modulus becomes increasingly large. The anisotropy of B_6_C_2_O_5_ in the shear modulus decreases first and then increases.

The 2D representations of the Young’s moduli of B_2_CO_2_ and B_6_C_2_O_5_ in the (001) plane (*xy* plane), (010) plane (*xz* plane), (100) plane (*yz* plane) and (111) plane are displayed in Figure 7a–h, respectively. The anisotropy of Young’s modulus at each plane is also different, taking B_2_CO_2_ as an example. The Young’s modulus in the (010) plane (*E*_max_/*E*_min_ = 628 GPa/414 GPa = 1.52) of B_2_CO_2_ exhibits the largest anisotropy compared to the other planes, while the (100) plane (*E*_max_/*E*_min_ = 734 GPa/571 GPa = 1.29) exhibits the smallest anisotropy. This result can also be demonstrated by the ratio of the maximum to the minimum of the Young’s modulus at each plane. The maximum values of Young’s modulus for B_2_CO_2_ appear in the (001) plane and (100) plane, while the minimum value of Young’s modulus appears in the (010) plane. The maximum and minimum values appear in the same plane, whether at ambient pressure or at high pressure. Another interesting phenomenon is that the anisotropy of Young’s modulus in the (001) plane decreases with increasing pressure, and that of the (010) plane increases with increasing pressure, while those of the (100) plane and (111) plane decrease first and then increase. For the whole material, the anisotropies of the Young’s moduli of B_2_CO_2_ and B_6_C_2_O_5_ decrease first and then increase. In addition, although the maximum and minimum values appear in the same plane, whether at ambient pressure or at high pressure, the direction is changed. For B_6_C_2_O_5_, the maximum value appears at θ = 2.82, φ = 5.93 (the two angles are used to describe the unit vector, which is fully characterized by the angles θ (0, π), φ (0, 2π), as explained in more detail in Refs. [48,49]) at ambient pressure, where the angle is in radians. While the maximum value appears at θ = 1.59, φ = 2.90 at 10 GPa, for the pressure of 20 GPa, the maximum value appears at θ = 1.55, φ = 2.73. At these three pressures (0 GPa, 10 GPa, 20 GPa), the direction of the minimum appears to be different. However, for B_2_CO_2_, the maximum values all appear at θ = 1.57, φ = 4.73, whether at ambient pressure or at high pressure, but the direction of the minimum appears to have changed.

### 3.4. Electronic Properties

**Figure 8:**
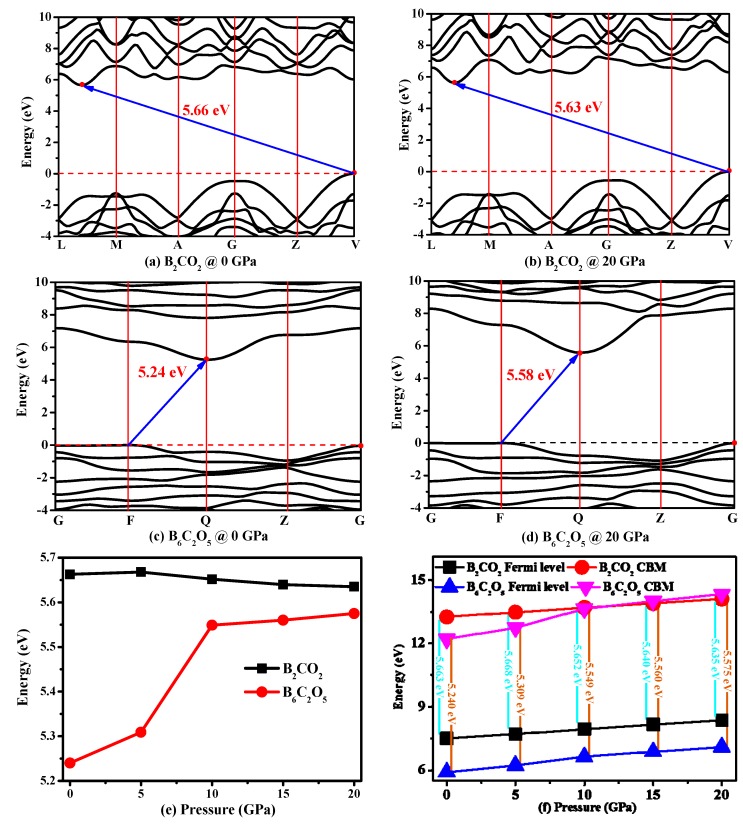
Band structures of: B_2_CO_2_ ((**a**): 0 GPa; and (**b**): 20 GPa); and B_6_C_2_O_5_ (**c**): 0 GPa; and (**d**): 20 GPa); band gaps as functions of pressure for B_2_CO_2_ and B_6_C_2_O_5_ (**e**); and Fermi level and CBM as functions of pressure for B_2_CO_2_ and B_6_C_2_O_5_ (**f**).

The electronic band structure is a significant physical property of a material that can be used to determine whether the material is a semiconductor, metal, or insulator. The electronic band structures of B_2_CO_2_ and B_6_C_2_O_5_ under different pressures are illustrated in Figure 8a–d, respectively. In Figure 8a–d, it is clear that B_2_CO_2_ and B_6_C_2_O_5_ are both indirect and wide band gap semiconductor materials with the band gaps of 5.63 and 5.24 eV, respectively. For B_6_C_2_O_5_, the conduction band minimum (CBM) and valence band maximum (VBM) are both located at the Dirac points in the Brillouin zone, the CBM of B_2_CO_2_ is also located at the Dirac point in the Brillouin zone, while the VBM of B_2_CO_2_ is not at the Dirac point in the Brillouin zone. The CBM and VBM are both located at the Q and F points in the Brillouin zone for B_6_C_2_O_5_, and the CBM of B_2_CO_2_ is also located at the V point in the Brillouin zone, while the VBM of B_2_CO_2_ is located at (−0.5000, −0.2143, 0.5000) along the L-M direction (L: (−0.5, 0.0, 0.5); M: (−0.5, −0.5, 0.5)) in the Brillouin zone. 

The band gaps of B_2_CO_2_ and B_6_C_2_O_5_ under different pressures are shown in Figure 8e. The two materials show different trends for the change of the band gap with increasing pressure. The band gap of B_6_C_2_O_5_ increases with pressure, while the band gap of B_2_CO_2_ first increases and then decreases. The change trend of the band gap of B_6_C_2_O_5_ is similar to that of diamond and c-BN [22]: the band gap of B_6_C_2_O_5_, diamond and c-BN all increase with increasing pressure, but the changes for the band gaps of diamond and c-BN are relatively smooth, and the band gap changes for B_6_C_2_O_5_ are quite abrupt. Then, we investigated the origin of the pressure-driven abrupt increase of the band and the non-monotonic trend of first increase and then decrease of the band gap for B_6_C_2_O_5_ and B_2_CO_2_, respectively. We analyzed the trend in the Fermi level and the conduction band minimum with the change of pressure. The relationships between the Fermi level and the conduction band minimum for B_2_CO_2_ and B_6_C_2_O_5_ and the pressure are shown in Figure 8f. For the Fermi level and the conduction band minimum of B_2_CO_2_ and the conduction band minimum of B_6_C_2_O_5_, the changes are relatively smooth, but the energy at the CBM of B_6_C_2_O_5_ increases abruptly from 0 GPa to 5 GPa and from 5 GPa to 10 GPa. Therefore, the band gaps of B_6_C_2_O_5_ changes abruptly due to the sudden increase in the energy at the CBM leading to a sudden increase of the band gap from 5.309 eV under 5 GPa to 5.549 eV under 10 GPa.

**Figure 9:**
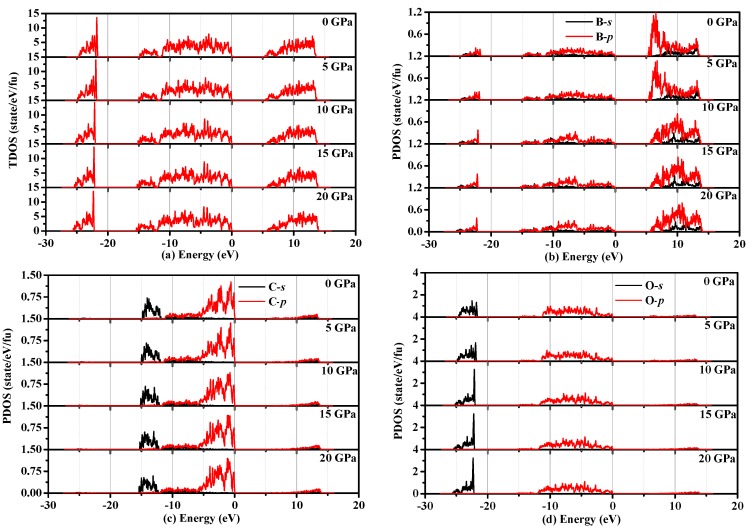
Density of states of B_6_C_2_O_5_ under different pressures: total density of states (**a**); partial density of states of B atom (**b**); partial density of states of C atom (**c**); and partial density of states of O atom (**d**).

To better understand why the band gap of B_6_C_2_O_5_ becomes larger with increased pressure, we also analyze the density of states of B_6_C_2_O_5_. The densities of states of B_6_C_2_O_5_ under different pressures are shown in Figure 9. Examination of Figure 9a shows that the valence band moves towards low energy, while the conduction band moves towards high energy in the 0–20 GPa range, so that the band gap of B_6_C_2_O_5_ increases with increased pressure. However, as shown in Figure 8e, the Fermi level increases with increased pressure, so, why does the band gap of B_6_C_2_O_5_ increase with the pressure in the 0–20 GPa range? According to Figure 9c, the density of states of the carbon atom almost does not vary with the pressure. Figure 9 b,d shows that, considering the density of states distribution of the oxygen atom, the peak value of the oxygen contribution to the conduction band increases with the increasing pressure, indicating that for the oxygen atoms under the action of external pressure, the local energy level is higher, resulting in reduced oxygen ions and leading to further changes of the band gap. Further consideration of the B atoms shows that the peak density of states increases with increasing pressure, decreasing the degree of localization of these atoms; however, the decrease in the density of states (from 1.07 state/eV/fu to 0.82 state/eV/fu when the pressure increases from 5 GPa to 10 GPa) is much smaller than the increase in the degree of the O atom localization (from 1.62 state/eV/fu to 3.24 state/eV/fu when the pressure increases from 5 GPa to 10 GPa), so that the results showed that the band gap of B_6_C_2_O_5_ becomes larger.

## 4. Conclusions

Based on first-principles calculations, we have systematically investigated two ternary light element compounds, B_6_C_2_O_5_ and B_2_CO_2_. We find that such two *sp*^2^ and *sp*^3^ hybridized structures could be obtained by other compounds and elements and that these structures are dynamically stable through calculations of the phonon spectra for B_6_C_2_O_5_ and B_2_CO_2_. The electronic band structures are also calculated, showing that B_2_CO_2_ and B_6_C_2_O_5_ are wide-gap semiconductor materials with indirect energy gaps of approximately 5.66 and 5.24 eV, respectively. The elastic anisotropy of B_2_CO_2_ and B_6_C_2_O_5_ phase has been demonstrated by the Young’s modulus and shear modulus along different crystal orientations. The elastic anisotropy results show that B_6_C_2_O_5_ exhibits a larger anisotropy in the shear modulus, while B_2_CO_2_ exhibits a larger anisotropy in Young’s modulus at ambient pressure. Another interesting phenomenon is that the anisotropy of the shear modulus of B_2_CO_2_ increases with increasing pressure, while the anisotropy of Young’s modulus of B_2_CO_2_, and the anisotropy of Young’s modulus and the shear modulus of B_6_C_2_O_5_ all first decrease and then increase with increasing pressure. The changes of the physical properties of B_2_CO_2_ and B_6_C_2_O_5_ show different trends with the pressure, and are strongly related to the crystal structure and atomic composition. The origin of band gap increases with increased pressure is explained from the viewpoint of the density of states. In addition, hardness calculations using the Lyakhov-Oganov model show that B_2_CO_2_ is a potential superhard material. 

## Figures and Tables

**Table 1:** Calculated lattice parameters of B_2_CO_2_, B_6_C_2_O_5_ and other B-C-O compounds.

Materials	Source	PBE				PBEsol				CA-PZ			
		*a* (Å)	*b* (Å)	*c* (Å)	*β* (°)	*a* (Å)	*b* (Å)	*c* (Å)	*β* (°)	*a* (Å)	*b* (Å)	*c* (Å)	*β* (°)
B_2_CO_2_	This work	9.773	2.487	5.395	90.9	9.721	2.479	5.368	90.8	9.606	2.456	5.308	90.9
	ref. [12]	9.776	2.489	5.395	90.8								
B_6_C_2_O_5_	This work	4.503	4.536	4.557	98.1	4.479	4.526	4.508	99.3	4.278	4.474	4.457	99.3
	ref. [12]	4.502	4.538	4.557	98.2								
tP4-B_2_CO	This work	2.656	2.656	3.678	90.0	2.648	2.648	3.656	90.0	2.618	2.618	3.619	90.0
	ref. [9]									2.623	2.623	3.623	90.0
tI16-B_2_CO	This work	3.722	3.722	7.493	90.0	3.702	3.702	7.515	90.0	3.664	3.664	7.376	90.0
	ref. [9]									3.670	7.394	7.394	90.0
B_2_C_2_O	This work	2.638		18.274	90.0	2.627		18.178	90.0	2.598		17.996	90.0
	ref. [10]	2.647		18.272	90.0								

**Table 2:** Calculated elastic constants (GPa) and elastic modulus (GPa) of B_2_CO_2_ and B_6_C_2_O_5_ under different pressures (*P*: in GPa).

Materials	*P*	*C*_11_	*C*_22_	*C*_33_	*C*_44_	*C*_55_	*C*_66_	*C*_12_	*C*_13_	*C*_23_	*C*_15_	*C*_25_	*C*_35_	*C*_46_	*B*	*G*	*E*
B_2_CO_2_	0	585	784	613	256	213	264	117	65	136	−77	13	51	−9	287	251	583
	5	622	813	649	267	221	269	125	82	145	−81	16	55	−10	306	260	608
	10	658	841	686	268	223	279	132	96	157	−85	15	57	−10	325	267	629
	15	694	872	718	274	226	281	141	110	168	−90	16	58	−13	344	273	648
	20	723	902	747	260	225	283	150	126	180	−95	13	64	−18	362	272	653
B_6_C_2_O_5_	0	528	519	571	207	170	211	102	40	83	6	−0.2	−72	−8	227	203	469
	5	549	547	596	216	163	215	108	35	93	−10	−1	−87	−10	234	207	480
	10	640	534	631	233	191	237	131	81	122	26	6	−44	5	274	224	528
	15	665	573	665	243	204	245	142	91	133	31	8	−49	7	293	234	554
	20	704	613	697	258	210	259	155	104	142	35	11	−53	9	312	244	581
tP4-B_2_CO	0 ^1^	736		591	240		254	53	157						311	254	
tI16-B_2_CO	0 ^1^	600		646	304		283	182	144						310	265	
B_2_C_2_O	0 ^2^	763		590	229		274	15	135						299	264	611
c-BN	0 ^3^	779			446			165							370	384	
*Pnma*-BN	0 ^4^	392	770	675	299	272	187	99	256	116					298	227	543
Diamond	0 ^3^	1053			563			120							431	522	1116

^1^ Reference [9]; ^2^ Reference [10]; ^3^ Reference [22]; ^4^ Reference [46].

**Table 3:** Calculated the maximum values (in GPa) and minimum values (in GPa) of Young’s modulus and shear modulus and the *X*_max_/*X*_min_ ratio for B_2_CO_2_ and B_6_C_2_O_5_.

Materials	*P*	0 GPa			10 GPa			20 GPa		
		Max	Min	Ratio	Max	Min	Ratio	Max	Min	Ratio
B_2_CO_2_	*G*	309	170	1.82	333	177	1.88	353	175	2.02
	*E*	734	414	1.77	785	446	1.76	837	460	1.82
B_6_C_2_O_5_	*G*	278	140	1.96	295	161	1.83	321	170	1.88
	*E*	603	345	1.75	657	408	1.61	717	431	1.66
